# Dietary carbohydrate intake and health-related outcomes: a protocol for the evidence evaluation methodology for the new guideline on dietary carbohydrate intake of the German nutrition society

**DOI:** 10.1007/s00394-025-03744-4

**Published:** 2025-06-23

**Authors:** Sabrina Schlesinger, Johanna Conrad, Anna Maria Amini, Anette Buyken, Sarah Egert, Julia Haardt, Nicole Kalotai, Anja Kroke, Lukas Schwingshackl, On behalf of the German Nutrition Society

**Affiliations:** 1 German Diabetes Center, Institute for Biometrics and Epidemiology, Düsseldorf, Germany; 2 German Center for Diabetes Research (DZD), Munich-Neuherberg, Partner Düsseldorf, Neuherberg, Germany; 3 German Nutrition Society, Bonn, Germany; 4 Institute of Nutrition, Consumption and Health, Faculty of Natural Sciences, Paderborn University, Paderborn, Germany; 5 Institute of Nutritional and Food Sciences, Nutritional Physiology, University of Bonn, Bonn, Germany; 6 Department of Nutritional, Food and Consumer Sciences, Fulda University of Applied Sciences, Fulda, Germany; 7 Institute for Evidence in Medicine, Medical Center, Faculty of Medicine, University of Freiburg, Freiburg, Germany

## Abstract

**Purpose:**

This protocol outlines the rigorous process for developing the new German guideline on dietary carbohydrate intake and its impact on health-related outcomes, building on a substantial body of evidence regarding carbohydrate quantity and quality.

**Methods:**

A professional guideline panel has been established, and research questions have been formulated a-priori. The novel methodological approach follows a structured process and adheres to established recommendations for guidelines.

**Results:**

Umbrella reviews of systematic reviews with meta-analyses of randomized controlled trials or prospective observational studies on carbohydrate quantity and quality in relation to health-related outcomes will be conducted. Relevant aspects of carbohydrate intake include total carbohydrates, dietary sugars, starch, the dietary glycaemic index, the dietary insulin index, dietary glycaemic load, dietary insulin load, dietary fibre, and whole grains. The prioritized outcomes include body weight-related outcomes, type 2 diabetes, dyslipidemia, elevated blood pressure, cardiovascular diseases, cancer, and dental caries and periodontal diseases. The literature search will be conducted in MEDLINE and Epistemonikos starting from 2015, as older reports are likely covered or superseded by more recent ones. The most comprehensive systematic review with meta-analysis with the highest methodological quality standards (according to a modified version of AMSTAR 2) will be selected for each specific association or effect. The certainty of evidence will be rated using the GRADE approach. The guideline development will follow the Evidence-to-Decision (EtD) framework.

**Conclusions:**

This methodological protocol is in line with international standards and provides transparent framework for the guideline on dietary carbohydrate intake and health-related outcomes of the Germany Nutrition Society.

**Supplementary Information:**

The online version contains supplementary material available at 10.1007/s00394-025-03744-4.

## Introduction

Public health decision-making in nutrition, including guideline development, should follow current international standards [1, 2]. These standards emphasise generating a body of evidence and rigorously evaluating the certainty of evidence. In this context, the use of the Grading of Recommendations Assessment, Development and Evaluation (GRADE) approach is mandatory [3], which also addresses research questions from non-randomized studies [4]. This is important because much of the evidence on dietary factors and health-related outcomes, particularly long-term disease risk, comes from prospective observational studies. To address broad public health questions, such as how dietary factors like carbohydrate intake influence human health, employing an umbrella review approach is a key innovation. This method allows for a broad systematic overview across a wide spectrum of exposures and health-related outcomes.

Moreover, final recommendations should not only consider the certainty of the evidence but also its public health implications. The Evidence to Decision (EtD) framework provides guidance for this purpose [5, 6]. Implementing this multi-step procedure depends on country-specific conditions, including the target audience, political and legal frameworks, and cultural factors. Thus, an a priori outline of the specific procedures is essential for developing a new guideline.

The German Nutrition Society (*Deutsche Gesellschaft für Ernährung e. V.*, DGE) regularly publishes nutritional guidelines on macronutrient intake based on meta-evidence linking macronutrient intake to the risk of health-related diseases, with the latest being the protein guideline [6]. Since the last guideline on dietary carbohydrate intake was published in 2011 [7], new evidence from systematic reviews and meta-analysis has emerged. A new guideline is therefore needed to reflect and synthesize this updated body of evidence. To achieve this, an umbrella review of systematic reviews and meta-analyses is warranted to provide a comprehensive overview of the findings and support the development of the new guideline. This new guideline will cover aspects of dietary carbohydrate quantity and quality in relation to selected health-related outcomes, including body weight-related outcomes, type 2 diabetes, dyslipidaemia, elevated blood pressure, cardiovascular diseases (CVD), cancer, dental caries and periodontal diseases.

The objective of this protocol is to outline the novel methodological procedure to be applied and to transparently describe each planned step for the new upcoming DGE guideline on dietary carbohydrate intake and health-related outcomes.

## Methods

### Scope

Since the DGE published its latest evidence-based guideline on carbohydrate intake and selected health-related outcomes in 2011 [7], a substantial body of evidence from systematic reviews and meta-analyses on this topic has become available. Consequently, a professional guideline panel has been established to develop a new guideline, comprising DGE staff members (AMA, JC, JH, NK), experts in evidence synthesis (LS, SS), and is coordinated by DGE scientific board members (AB, AK, SE). The research questions were postulated a-priori and the exposures and outcomes were selected by discussion with experts and consensus-based decision by the panel.

This protocol describes each step of the methodological approach to ensure transparency in evidence synthesis and the application of the decision framework. The methodological procedure has been registered at PROSPERO (CRD42025589418). All steps of this protocol will be reported in accordance with the PRISMA guideline [8] and its extension for protocols [9], as well as with the PRIOR statement [10], the reporting check lists for guidelines [11], and the WHO handbook for guideline development [12].

### Study design

Umbrella reviews will be performed for this new evidence-based guideline on carbohydrate intake and selected health-related outcomes. This involves conducting a systematic review of systematic reviews with meta-analyses, considering evidence from both randomized controlled trials (RCTs) and observational prospective studies, as RCTs are often not feasible for investigating the relevance of dietary factors on long-term disease risk.

### Eligibility criteria

Systematic reviews with meta-analysis investigating the association or the effect of carbohydrates, including quantity and quality of carbohydrates and its subtypes, on selected health-related outcomes, including body weight-related outcomes, type 2 diabetes, dyslipidaemia, elevated blood pressure, CVD, cancer, and dental caries and periodontal diseases will be eligible. The PICOS/PECOS scheme is summarized in Table 1.

**Table 1:** PICOS/PECOS table for study selection: inclusion and exclusion criteria

	Inclusion	Exclusion
**P**	Generally healthy population, focusing on adults; children and adolescents are only considered for body weight-related outcomes and dental caries	Studies including *only* pregnant or breastfeeding women, top athletes, individuals with chronic conditions (e.g., type 2 diabetes, obesity, cardiovascular disease, cancer survivors), individuals undergoing therapy; For outcomes *other than* body weight-related outcomes, dental caries and periodontal diseases: children and adolescents
**I/E**	Quantity and quality of carbohydrates; see Table 2	Other than reported in Table 2
**C**	High vs. low comparisons, dose-response associations, substitution effects; for RCTs: placebo, controls, standard of care	Not applicable
**O**	Health-related outcomes; see Table 3	Other than reported in Table 3
**S**	Systematic reviews with meta-analysis of RCTs (intervention duration ≥ 4 weeks) or prospective observational studies	Systematic reviews without meta-analysis, meta-analyses without systematic reviews, umbrella reviews, pooled analyses, systematic reviews with meta-analyses on cross-sectional or retrospective case-control studies, systematic reviews with meta-analyses of RCTs with intervention duration < 4 weeks, and single studies

*Population*: The population of interest will be adults (aged ≥ 18 years) from the general population. This excludes studies focusing solely on patient populations (e.g., persons with type 2 diabetes, obesity, CVD, chronic kidney diseases or cancer), competitive athletes, as well as pregnant or lactating women. For body weight-related outcomes, dental caries and periodontal diseases, systematic reviews and meta-analyses involving children and adolescents will additionally be included. This decision acknowledges that these conditions develop already among younger populations, whereas conditions such as type 2 diabetes, CVD, and cancer do typically not emerge in these age groups.

*Exposure/Intervention*: Our umbrella reviews will comprehensively explore a wide range of dietary carbohydrate intake aspects, considering both quantity and quality, as well as their possible combinations (e.g. carbohydrate intake from low GI sources). This includes total carbohydrates, total sugar, added sugar, free sugar, specific sugars (e.g., glucose and fructose), sugar-sweetened beverages, juices, and other non-alcoholic drinks with added sugar (like coffee or tea) [13]. We will also focus on whole grains, various types of fibre (soluble, insoluble), fibre from different sources like cereal, fruit, vegetables, nuts, and seeds, and starch. Additionally, we will evaluate the dietary glycaemic index, glycaemic load, insulin index, and insulin loads, as dietary exposures. All eligible exposures are detailed in Table 2. Depending on the outcome and the investigated associations, some or all of the listed exposures will be addressed.

**Table 2:** Exposures/interventions of interest

Exposure/Intervention	Details
**Total carbohydrate intake**	
**Sugar intake**	Total sugar Added sugar Free sugar Single sugars (glucose, fructose), Sugar-sweetened beverage, juices, and other non-alcoholic drinks with added sugars (such as coffee and tea) Note: Biomarker studies are also taken into account.
**Starch intake**	Total Starch Resistant and non-resistant starch
**Metrics related to carbohydrates**	Dietary glycaemic index, dietary glycaemic load, dietary insulin index, dietary insulin load
**Dietary fibre intake**	Total fibre Soluble and insoluble Dietary fibre from different sources (cereals, vegetable, fruit, legumes, nuts and seeds)
**Whole grain intake**	Total whole grain intake Whole grain products Note: Biomarker studies are also taken into account.

*Comparators*: Eligible systematic reviews with meta-analysis have to report one of the following: a high versus low intake of the described exposures, a dose-response association, a modelled substitution effect of the exposure by other nutrients (or, in the case of sugar-sweetened beverages/juice/non-alcoholic drinks with added sugars, substitution by other non-alcoholic drinks, including artificially sweetened beverages), or a comparison with a placebo or control diet in RCTs. If a systematic review with meta-analysis reports both high vs. low comparisons and dose-response associations, priority will be given to the dose-response analysis. High vs. low comparisons will only be considered when dose-response data are not available. Additionally, substitution meta-analyses will also be considered.

*Outcomes*: The selected outcomes are shown in Table 3. First, we will focus on body weight-related outcomes, including general overweight and obesity (defined by body mass index (BMI) cut-offs), abdominal overweight and obesity (defined by waist circumference (WC) cut-offs), BMI/BMI change, body weight/body weight change, WC/WC change, waist-to-hip-ratio/waist-to-hip-ratio change, fat mass/fat mass change (in kg and %), fat free mass/fat free mass change (in kg and %),fat mass index/fat mass index change, fat free mass index/fat free mass index change, visceral fat, liver fat (content), non-alcoholic fatty liver disease (NAFLD)/metabolic dysfunction-associated steatotic liver disease (MASLD). For children and adolescents, we also include body weight-related outcomes reported as standard deviation scores (SDS) or z-scores. Second, a further outcome of interest includes the incidence of type 2 diabetes. Third, systematic reviews with meta-analysis on incidence of dyslipidemia and the following blood markers will be eligible: total cholesterol, low-density lipoprotein cholesterol (LDL-C), high-density lipoprotein cholesterol (HDL-C), non-HDL-C, triglycerides, lipoprotein(a), apolipoprotein A1 (ApoA1), apolipoprotein B (ApoB). Fourth, for blood pressure, we consider incidence of hypertension, systolic and diastolic blood pressure, as well as ambulatory blood pressure measurements (ABPM) / parameters of the blood pressure monitoring. Fifth, CVD incidence and CVD mortality will be included as further outcome. This incorporates total CVD, coronary heart disease, myocardial infarction, stroke (ischemic stroke and hemorrhagic stroke), and heart failure. Sixth, cancer incidence and cancer mortality will be included, considering total cancer and specific sites, e.g., colorectal, breast, and prostate cancer. Seventh, we will also include systematic reviews with meta-analyses focusing on incidence of dental caries (including decayed, missing, and filled teeth) and incidence of periodontal diseases (including periodontitis and gingivitis). Other outcomes not listed here will not be addressed.

**Table 3:** Selected outcomes

Outcome	Details
**Body weight-related outcomes**	General overweight and obesity, abdominal overweight and obesity, BMI/BMI change, body weight/body weight change,, waist circumference/ waist circumference change, waist-to-hip-ratio/waist-to-hip-ratio change, fat mass/change of fat mass (kg and %), fat free mass/change of fat free mass (kg and %), fat mass index/change of fat mass index, fat free mass index/fat free mass index change, visceral fat, liver fat (content), NAFLD, MASLD, BMI-SDS and z-scores for children and adolescents
**Type 2 diabetes**	Incidence of type 2 diabetes
**Dyslipidaemia**	Incidence of dyslipidaemia, levels of total cholesterol, low-density lipoprotein cholesterol (LDL-C), high-density lipoprotein cholesterol (HDL-C), non-HDL-C, triglycerides, lipoprotein(a) [lp(a)], apolipoprotein A1 (ApoA1), apolipoprotein B (ApoB)
**Hypertension**	Incidence of hypertension, systolic and diastolic blood pressure, ambulatory blood pressure measurements (ABPM), ambulatory blood pressure monitoring
**CVD**	CVD incidence and mortality, including total CVD, coronary heart disease, myocardial infarction, stroke (ischemic stroke and hemorrhagic stroke), and heart failure
**Cancer**	Total cancer incidence and cancer mortality, and different cancer sites (e.g., colorectal cancer, breast cancer, prostate cancer)
**Dental caries and periodontal diseases**	Incidence of dental caries (including decayed, missing, and filled teeth), and incidence of periodontal diseases (including periodontitis and gingivitis)

*Study design*: Systematic reviews with pairwise, linear and non-linear dose-response, substitution or network meta-analyses on longer-term RCTs ) or prospective observational studies will be eligible for inclusion. The minimum intervention duration will generally be set at ≥ 4 weeks. However, for specific outcomes such as blood lipids, a shorter duration of ≥ 2 weeks will be accepted due to the known responsiveness of these markers to dietary changes. Systematic reviews without a meta-analysis, pooled studies without a systematic review, umbrella reviews, or single study findings will be excluded. In addition, systematic reviews and meta-analysis including cross-sectional or retrospective case-control studies will not be eligible for inclusion. However, if subgroup meta-analyses for prospective studies are available, the article will be considered for inclusion. Systematic reviews and meta-analysis of RCTs on shorter-term effects will be excluded as well.

### Search strategy

The systematic literature search will be conducted in MEDLINE and Epistemonikos. An experienced information specialist has designed the search term for each database, and an example for MEDLINE is shown in the Supplement Table 1. The search will be limited to articles published from 2015 onward, as older systematic reviews with meta-analyses are likely covered or superseded by more recent ones [14]. During the search, we will not apply any filters or restrictions, such as the human or language filter in MEDLINE. To identify further relevant articles, we will implement backward and forward snowballing, which involves searching the reference lists of included studies as well as searching further studies that have cited them.

### Screening and study selection

The identified records from the databases will be downloaded and merged into a single database in EndNote, where duplicates will be identified and removed. This database will then be uploaded to Covidence, where remaining duplicates will be automatically identified and removed. The screening of titles and abstracts, as well as the selection of studies after reading the full texts, will be independently conducted by pairs of investigators. Based on predefined inclusion and exclusion criteria, investigators will determine the eligibility of each study. Any discrepancies will be resolved through consensus between the investigators or by consulting a third investigator. If an article is identified that is not available in English or German, we will use a web-based tool for translation (e.g., DeepL https://www.deepl.com). A list of excluded records after full-text assessment including justifications for exclusion will be provided.

We will include the most comprehensive systematic review with meta-analysis that meets the highest methodological standards to avoid overlap of primary studies across multiple systematic reviews on the same topic [15]. To identify the most suitable report, we will first select the three reports with the largest number of included studies. We will then assess their quality using the critical appraisal tool, ‘A MeaSurement Tool to Assess systematic Reviews’ (AMSTAR 2). Any reviews rated as “critically low” will be excluded, and the report with the highest methodological quality rating will be selected for inclusion. In case of equal ratings, the report with the largest number of included studies will be selected. Conflicting findings between meta-analyses will be discussed and contextualized accordingly.

### Data extraction

We will use a pilot-tested Excel extraction sheet for data extraction. One investigator will conduct the data extraction, and a second independent investigator will double-check the extracted data for accuracy. Any discrepancies will be resolved through discussion or by consulting an experienced methodologist within the guideline group.

The following data– if reported– will be extracted from the systematic review with meta-analysis: first author’s last name, year of publication, type of meta-analysis (pairwise, linear dose-response, non-linear dose-response, substitution meta-analysis, or network meta-analysis), study design of the primary studies (RCTs or prospective observational studies), study period of the included primary studies, study population, exposure(s)/intervention(s), dietary assessment method to derive information on carbohydrates (exposure)/information on delivery of the intervention(s) and its duration, dose of exposure intake/intervention dose (i.e. intake level of the respective carbohydrate parameter), if applicable comparator, control group, or substitute of the exposure, indication for non-linearity (including clinically relevant thresholds) for non-linear dose-response meta-analysis, outcome(s), method for outcome measurement, the summarised effect estimate (Hazard Ratios (HR), Relative Risks (RR), Odds Ratios (OR), mean differences) including 95% confidence interval (CI) for each exposure/intervention association/effect for/on each outcome, considered confounders, findings from sensitivity and subgroups meta-analyses (e.g., adjustment with and without energy intake or BMI, or adjustments for dietary fibre or macronutrients (e.g. protein intake)), measures of heterogeneity and inconsistency, indication for publication bias, and assessments and results of the evaluation of the risk of bias (Cochrane RoB 2 tool [16], ROBINS-I/E [17, 18], the certainty of evidence [3]), the funding sources of the primary studies and the funding sources/conflict of interests of the authors of the systematic review.

### Assessment of the methodological quality

The methodological quality of the selected systematic review with meta-analysis, will be assessed with a modified version of the AMSTAR 2 guidance [19]. This rating will be conducted by two independent investigators and classified as high, moderate, low or critically low methodological quality according to the existence of critical and non-critical methodological weaknesses. The slight modifications to the instrument were implemented based on the approach described previously [6]). Key modifications to AMSTAR 2 included reclassifying some items as non-critical domains (e.g., registration of study protocols, risk of bias consideration) and excluding specific items/sub-items (e.g., searching gray literature, consulting content experts). Additionally, some criteria were adapted to better align with the umbrella review format, such as simplifying “partial yes” responses, focusing on quality score system to assess risk of bias, and excluding requirements that were irrelevant for the research aim. The modified version is described in detail in the Supplement Table 2.

### Evaluation of the certainty of evidence (CoE) for each outcome

Each pooled effect estimate with 95% CI for each exposure/intervention on each outcome, will be evaluated separately by two independent investigators using the GRADE approach [3]. The findings will be shown in an evidence profile. The CoE can be classified as high, moderate, low or very low. Regarding observational studies, we will follow predefined procedures: (I) if a systematic review with meta-analysis assessed the risk of bias by applying established instruments such as the recently published ROBINS-E [18] or also the longer-available ROBINS-I tool [17], the study will also start at a high level of CoE and will undergo the procedure similar to RCTs [4], (II) if the study did not assess the risk of bias or did not apply the mentioned tools but used quality scores instead, certainty assessment will start with a low CoE. The GRADE approach consists of five downgrading domains (risk of bias, indirectness, inconsistency, imprecision, publication bias) and three upgrading domains (large magnitude of effect, dose-response gradient, plausible confounding [this domain is only relevant if certainty start at low CoE]).

### Biological plausibility

The experts will check whether biological plausibility can be assumed. The biological plausibility is considered if findings from prospective observational studies can be explained by a biological effect model (e.g., cell culture and/or animal model study) and/or findings can be demonstrated in controlled human dietary intervention studies. Additionally, biological plausibility will be integrated into the ‘indirectness’ domain of the GRADE approach. Specifically, if an association or effect is demonstrated but is biologically implausible, we will downgrade the certainty of evidence by one level [20].

### Summary of results and derivation of the recommendation

For each predefined outcome (Table 3), a guideline chapter will be prepared and submitted for publication in an international peer-reviewed journal. After finalising the single outcome chapters, the results will be summarised and the evidence for the GRADE EtD criteria will be collected [5, 6]. The DGE staff members will handle the GRADE assessment and prepare the GRADE EtDs. Following the EtD principles, they will address additional criteria using the best available evidence, documenting both evidence and judgments. The panel will consider the following criteria, supported by the best available evidence:

1. Certainty of evidence.
2. Problem priority: prioritising outcomes based on epidemiological data, including their prevalence and disease severity indicators. This will be evaluated based on disability-adjusted life years, their burden ranking, disease costs, and the influence of nutrition on the outcomes, as well as the clinical relevance [21]. Outcomes with higher prevalence, greater disease severity, and higher associated costs will be prioritised. Clinically relevant outcomes, such as CVD, type 2 diabetes, and cancer, will have a stronger impact on the overall recommendations than outcomes based solely on surrogate markers like LDL cholesterol or ApoB. (In)consistency between findings on clinically relevant outcomes and surrogate markers will also be taken into account.
3. Weighing benefits and harms: balancing beneficial and harmful effects.
4. Dietary intake levels and preferences: considering dietary habits and cultural preferences.
5. Ecological sustainability: assessing environmental impact.
6. Acceptability and feasibility: evaluating implementation impact and potential barriers.
7. Potential impacts on health equity: considering effects on disadvantaged populations. This includes identification of potentially vulnerable groups regarding the quality or origin of the carbohydrates consumed and/or food insecurity as well as a narrative synthesis of evidence on the influence of the socioeconomic status on consumption pattern.

Based on this data, the guideline panel will derive consensus-based qualitative weighted overall recommendations. This includes decisions on the direction (for, against, or no recommendation) and strength (strong or conditional) of each recommendation, providing justifications [6].

### Draft, public consultation, and final publication of the guideline

A preliminary version of the guideline on dietary carbohydrate intake and health-related outcomes will be released online for public consultation. Feedback will be invited within a two-month period. After careful review and consideration of all comments by the guideline panel, the final version of the guideline will be published. Regarding conflict of interests, we have requested a declaration of interest from all protocol group members and provided respective declarations at the end of the manuscript. Moreover, we will request a declaration of interest from all guideline members and experts on the outcome-specific chapters, and make the respective declarations available on the DGE website.

## Discussion

This protocol outlines the process for the new dietary carbohydrate guideline of the DGE following an innovative approach, and the current international standards. It provides a transparent, step-by-step approach, from conducting umbrella reviews on carbohydrate quantity and quality to develop recommendations for the dietary guideline.

This new guideline on carbohydrates aims to serve as a comprehensive resource that informs public health policy, thereby supporting the nutritional well-being of the general population. A transparent process based on a rigorous systematic review of the literature, guided by the principles of evidence-based practice is an important prerequisite for the use of the guideline by policy makers and stakeholders involved in primary prevention. By disseminating our findings in outcome-specific chapters in peer-reviewed journals and by presenting the results at relevant conferences and professional meetings, we will also ensure that our findings advance the field of nutrition science. In addition, the findings will be provided for the public through a variety of channels, including a summary in German, dissemination via social media, integration into training courses and by informing the reference values of the DGE and the Austrian Nutrition Society (ÖGE), and food-based dietary guidelines.

Previous reports have addressed selected relations between carbohydrate intake and health-related outcomes [22–25]. However, these projects differ from ours. A key innovation of our project is the use of the broad umbrella review methodology, selecting the most comprehensive reports that simultaneously meet rigorous methodological standards. This approach allows for a broad(er), systematic overview across a wide(r) range of exposures related to dietary carbohydrate intake and health-related outcomes. Moreover, not all previous reports have considered dose-response, substitution and/or network meta-analyses, or applied the GRADE approach. Another innovative aspect of our work is the integration of the results into dietary guidelines using the EtD framework. Moreover, the DGE recently implemented a similar approach for generating the dietary protein guideline [6] with regard to health-related outcomes [26–29]. While the methodological approach is largely similar, several specific alterations have been integrated here. Compared to the protein guideline, the carbohydrate guideline will include only one systematic review with meta-analysis for each specific research question (e.g., same exposure and same outcome), considering both the largest number of studies and, simultaneously, the highest methodological quality, as assessed by a modified AMSTAR 2 tool.

The strengths of this protocol include the clear and transparent description of the procedure for this guideline on dietary carbohydrate intake. The guideline panel consist of diverse experts and involve further experts on the outcome-specific chapters. The methodology follows internationally accepted and established guidelines. This includes the rigorous literature search by an information specialist, the screening of the articles, data extraction, risk of bias assessment, and the evaluation of the CoE by at least two independent investigators. Moreover, the GRADE EtD framework will be used to translate findings into a guideline.

There are also some limitations that need to be discussed. According to this protocol only systematic reviews with meta-analysis will be included. Potentially relevant studies, not included in a systematic review and/or meta-analysis could be missed and not integrated in the guideline. The umbrella reviews will rely on published studies, and one limitation is the nature of the primary studies, which mostly include prospective observational study designs for several outcomes. This may raise challenges upon drawing conclusions about causality due to potential confounding factors and reporting biases.

In conclusion, this protocol will guide the development of the upcoming dietary carbohydrate guideline by the DGE. By employing umbrella reviews, we will ensure a comprehensive and systematic evaluation of the current evidence on how carbohydrate quantity and quality are linked to selected health-related outcomes. The assessment of the CoE by the GRADE tool, integrated into the GRADE EtD framework, will direct the development of an evidence-based dietary guideline.

## Electronic supplementary material

Below is the link to the electronic supplementary material.

Supplementary Material 1
