# Supplementary material for: Dietary carbohydrate intake and health-related outcomes: a protocol for the evidence evaluation methodology for the new guideline on dietary carbohydrate intake of the German nutrition society
Source: Eur J Nutr. 2025 Jun 23;64(5):226. doi: 10.1007/s00394-025-03744-4 (PMC12185597; doi:10.1007/s00394-025-03744-4)
Supplement: Supplementary file 1 — Supplementary Material 1 [file 394_2025_3744_MOESM1_ESM.docx]

**Electronic Supplementary Material**

**Dietary Carbohydrate Intake and Health-Related Outcomes:**

**A Protocol for the Evidence Evaluation Methodology for the Guideline on dietary carbohydrate intake of the German Nutrition Society**

Sabrina Schlesinger,^1,2^ Johanna Conrad,^3^ Anna Maria Amini,^3^ Anette Buyken,^4^ Sarah Egert,^5^ Julia Haardt^3^, Nicole Kalotai,^3^ Anja Kroke,^6^ Lukas Schwingshackl ^7^ on behalf of the German Nutrition Society

^1^ German Diabetes Center, Institute for Biometrics and Epidemiology, Düsseldorf, Germany.

^2^ German Center for Diabetes Research (DZD), Munich-Neuherberg, Partner Düsseldorf, Germany.

^3^ German Nutrition Society, Bonn, Germany.

^4^ Institute of Nutrition, Consumption and Health, Faculty of Natural Sciences, Paderborn University, Paderborn, Germany.

^5^ Institute of Nutritional and Food Sciences, Nutritional Physiology, University of Bonn, Bonn, Germany.

^6^ Department of Nutritional, Food and Consumer Sciences, Fulda University of Applied Sciences, Fulda, Germany.

^7^ Institute for Evidence in Medicine, Medical Center and Faculty of Medicine, University of Freiburg, Freiburg, Germany.

Corresponding author: PD Dr. Sabrina Schlesinger, German Diabetes Center, Institute for Biometrics and Epidemiology, Düsseldorf, Germany. Email: corresponding_author@dge.de

**Supplementary Table S1: Search terms**

| (  "Dietary Carbohydrates"[**Mesh**] OR  Carbohydrate*[tiab] OR  "Sugars"[**Mesh**] OR  Sugar*[tiab] OR  Monosaccharide*[tiab] OR  Disaccharide*[tiab] OR  saccharose[tiab] OR  sucrose[tiab] OR  fructose[tiab] OR  glucose[tiab] OR  "Polysaccharides"[**Mesh**] OR  Polysaccharide*[tiab] OR  "Sugar-Sweetened Beverages"[**Mesh**] OR  ((sweetened[tiab] OR diet[tiab]) **AND** (beverage*[tiab] OR drink*[tiab] OR soda*[tiab] OR coffee*[tiab] OR tea[tiab] OR teas[tiab])) OR  “Soft drink*”[tiab] OR  “Energy Drinks”[**Mesh**] OR  “energy drink*”[tiab] OR  “Sport* drink*”[tiab] OR  “Fruit and Vegetable Juices”[**Mesh**] OR  juice*[tiab] OR  “fruit drink*”[tiab] OR  Fiber*[tiab] OR  Fibre*[tiab] OR  "Whole Grains"[**Mesh**] OR  "Whole Grain*"[tiab] OR  "Wholegrain*"[tiab] OR  Starch[tiab] OR  "Glycemic Index"[**Mesh**] OR  "glycemic ind*"[tiab] OR  "glycaemic ind*"[tiab] OR  "Glycemic Load"[Mesh] OR  "Glycemic Load"[tiab] OR  "Glycaemic Load"[tiab] OR “insulin index*”[tiab] OR “insulin load”[tiab]  )  AND |
| --- |
| 2015:2024[dp] |
| AND  (  Systematic[sb] **AND**  ("Meta-Analysis as Topic"[Mesh] OR  "Meta-Analysis"[Publication Type] OR  meta-analysis[tiab])  )  ) |

**Supplementary Table S2: Modified version of the ‘A MeaSurement Tool to Assess systematic Reviews’ (AMSTAR 2) tool**

| 1. **Did the research questions and inclusion criteria for the review include the components of PICO?** | | | | | | | | |
| --- | --- | --- | --- | --- | --- | --- | --- | --- |
| For Yes, all the following: | | | | | | |  | Yes |
|  |  |  |  |  |  |  |  | No |
|  | | Population | | | | |  | |
|  | | Intervention | | | | |  | |
|  | | Comparator group | | | | |  | |
|  | | Outcome | | | | |  | |
| 1. **Did the report of the review contain an explicit statement that the review methods were established prior to the conduct of the review and did the report justify any significant deviations from the protocol?** | | | | | | | | |
| For Partial Yes:  The authors state that they had a written protocol or guide that included all the following: | | | | | For Yes:  As for partial yes, plus the protocol should be registered and should also have specified: | |  | Yes |
|  |  |  |  |  |  |  |  | Partial Yes |
|  |  |  |  |  |  |  |  | No |
|  | review question(s) | | | |  | a meta-analysis/synthesis plan, if appropriate, *and* |  | |
|  | a search strategy | | | |  | a plan for investigating causes of heterogeneity |  | |
|  | inclusion/exclusion criteria | | | |  | justification for any deviations from the protocol |  | |
|  | a risk of bias assessment | | | |  |  |  | |
| 1. **Did the review authors use a comprehensive literature search strategy?** | | | | | | | | |
| For Partial Yes, all the following: | | | | | For Yes, should also have: | |  | Yes |
|  |  |  |  |  |  |  |  | Partial Yes |
|  |  |  |  |  |  |  |  | No |
|  | searched at least 2 databases (relevant to research question) | | | |  | searched the studies reference lists/bibliographies of included |  | |
|  | provided key word and/or search strategy | | | |  |  |  | |
| 1. **Did the review authors perform study selection in duplicate?** | | | | | | | | |
| For Yes, either one of the following: | | | | | | |  | Yes |
|  |  |  |  |  |  |  |  | No |
|  | at least two reviewers independently agreed on selection of eligible studies and achieved consensus on which studies to include | | | | | |  | |
|  | OR two reviewers selected a sample of eligible studies and achieved good agreement (at least 80 percent), with the remainder selected by one reviewer | | | | | |  | |
| 1. **Did the review authors perform data extraction in duplicate?** | | | | | | | | |
| For Yes, either ONE of the following: | | | | | | |  | Yes |
|  |  |  |  |  |  |  |  | No |
|  | at least two reviewers achieved consensus on which data to extract from included studies | | | | | |  | |
|  | OR two reviewers extracted data from a sample of eligible studies and achieved good agreement (at least 80 per cent), with the remainder extracted by one reviewer | | | | | |  | |
| 1. **Did the review authors provide a list of excluded studies and justify the**   **exclusions?** | | | | | | | | |
| For partial Yes: | | | | | For Yes, must also have: | |  | Yes |
|  |  |  |  |  |  |  |  | Partial Yes |
|  |  |  |  |  |  |  |  | No |
|  | provided a flow chart showing the number of excluded studies an reasons for exclusion. A study-specific list is not required | | | |  | provided a study-specific list and justified the exclusion from the review of each potentially relevant study |  | |
| 1. **Did the review authors describe the included studies in adequate detail?** | | | | | | | | |
| For Yes (all the following): | | | | | | |  | Yes |
|  |  |  |  |  |  |  |  | No |
|  | described populations | | | | | |  | |
|  | described interventions | | | | | |  | |
|  | described comparators | | | | | |  | |
|  | described outcomes | | | | | |  | |
|  | described research designs | | | | | |  | |
| 1. **Did the review authors use a satisfactory technique for assessing the risk of bias (RoB) in individual studies that were included in the review?** | | | | | | | | |
| For partial Yes: | | | | For Yes: | | |  | Yes |
|  |  |  |  |  |  |  |  | Partial Yes |
|  |  |  |  |  |  |  |  | No |
|  | A quality score system (e. g., NOS) was used to evaluate the risk of bias of included studies | | |  | | An established risk of bias tool (e.g., Cochrane risk of bias tool, ROBINS-I/E, Rob2.0) was used to evaluate the risk of bias of included studies |  | |
| 1. **If meta-analysis was performed did the review authors use appropriate methods for statistical combination of results?** | | | | | | | | |
| **RCTs** For Yes: | | | | | | |  | Yes |
|  |  |  |  |  |  |  |  | No |
|  | The authors justified combining the data in a meta-analysis | | | | | |  | |
|  |  |  |  |  |  |  |  | |
|  |  | | AND they used an appropriate weighted technique to combine study results and adjusted for heterogeneity if present (application of the random-effect model) | | | |  | |
|  |  | | AND investigated the causes of any heterogeneity in subgroup or sensitivity analysis | | | |  | |
| **NRSI**  For Yes: | | | | | | |  | Yes |
|  |  |  |  |  |  |  |  | No |
|  | The authors justified combining the data in a meta-analysis | | | | | |  | |
|  |  | | AND they used an appropriate weighted technique to combine study results, adjusting for heterogeneity if present (application of the random-effect model) | | | |  | |
|  |  | | AND they statistically combined effect estimates from NRSI that were adjusted for confounding, rather than combining raw data, or justified combining raw data when adjusted effect estimates were not available | | | |  | |
|  |  | | AND investigated the causes of any heterogeneity in subgroup or sensitivity analysis | | | |  | |
|  |  | | AND they reported separate summary estimates for RCTs and NRSI separately when both were included in the review | | | |  | |
| 1. **Did the review authors account for RoB in individual studies when interpreting/discussing the results of the review?** | | | | | | | | |
| For Yes: | | | | | | |  | Yes |
|  |  |  |  |  |  |  |  | No |
|  | included only studies with low risk of bias | | | | | |  | |
|  | OR risk of bias was considered in the discussion and interpretation | | | | | |  | |
| 1. **Did the review authors provide a satisfactory explanation for, and discussion of, any heterogeneity observed in the results of the review?** | | | | | | | | |
| For Yes: | | | | | | |  | Yes |
|  |  |  |  |  |  |  |  | No |
|  | There was no significant heterogeneity in the results | | | | | |  | |
|  | OR if heterogeneity was present the authors performed an investigation of sources of any heterogeneity in the results and discussed the impact of this on the results of the review | | | | | |  | |
| 1. **If they performed quantitative synthesis did the review authors carry out an adequate investigation of publication bias (small study bias) and discuss its likely impact on the results of the review?** | | | | | | | | |
| For Yes: | | | | | | |  | Yes |
|  |  |  |  |  |  |  |  | No |
|  | performed graphical or statistical tests for publication bias and discussed the likelihood and magnitude of impact of publication bias | | | | | |  | |
| 1. **Did the review authors report any potential sources of conflict of interest, including any funding they received for conducting the review?** | | | | | | | | |
| For Yes: | | | | | | |  | Yes |
|  |  |  |  |  |  |  |  | No |
|  | The authors reported no competing interests OR | | | | | |  | |
|  | The authors described their funding sources and how they managed potential conflicts of interest | | | | | |  | |
| **Overall rating**  Critical weakness(es):  Non-critical weakness(es):  Methodological quality of the review:  (high rating = no critical weakness with no or one non-critical weakness, moderate rating = no critical weakness with more than one non-critical weakness, low rating = one critical weakness with or without non-critical weaknesses, critically low rating = more than one critical weakness with or without non-critical weaknesses) | | | | | | | | |
| Note: This table provides a modified version of the AMSTAR 2 tool that will be used to assess the methodological quality of systematic reviews in the carbohydrate guideline. Please refer to the following publication for the original AMSTAR 2 tool: Shea BJ, Reeves BC, Wells G, Thuku M, Hamel C, Moran J, Moher D, Tugwell P, Welch V, Kristjansson E, Henry DA: AMSTAR 2: a critical appraisal tool for systematic reviews that include randomised or non-randomised studies of healthcare interventions, or both. BMJ 2017;358:j4008. | | | | | | | | |
